# Sulforaphane reactivates cellular antioxidant defense by inducing Nrf2/ARE/Prdx6 activity during aging and oxidative stress

**DOI:** 10.1038/s41598-017-14520-8

**Published:** 2017-10-26

**Authors:** Eri Kubo, Bhavana Chhunchha, Prerna Singh, Hiroshi Sasaki, Dhirendra P. Singh

**Affiliations:** 1 Department of Ophthalmology, Kanazawa Medical University, Kanazawa, Japan; 2 Department of Ophthalmology and Visual Science, University of Nebraska Medical Center, NE Omaha, USA

## Abstract

Upon oxidative stress and aging, Nrf2 (NFE2-related factor2) triggers antioxidant defense genes to defends against homeostatic failure. Using human(h) or rat(r) lens epithelial cells (LECs) and aging human lenses, we showed that a progressive increase in oxidative load during aging was linked to a decline in Prdx6 expression. DNA binding experiments using gel-shift and ChIP assays demonstrated a progressive reduction in Nrf2/ARE binding (−357/−349) of Prdx6 promoter. The promoter (−918) with ARE showed a marked reduction in young vs aged hLECs, which was directly correlated to decreased Nrf2/ARE binding. A Nrf2 activator, Sulforaphane (SFN), augmented Prdx6, catalase and GST*π* expression in dose-dependent fashion, and halted Nrf2 dysregulation of these antioxidants. SFN reinforced Nrf2/DNA binding and increased promoter activities by enhancing expression and facilitating Nrf2 translocalization in nucleus. Conversely, promoter mutated at ARE site did not respond to SFN, validating the SFN-mediated restoration of Nrf2/ARE signaling. Furthermore, SFN rescued cells from UVB-induced toxicity in dose-dependent fashion, which was consistent with SFN’s dose-dependent activation of Nrf2/ARE interaction. Importantly, knockdown of Prdx6 revealed that Prdx6 expression was prerequisite for SFN-mediated cytoprotection. Collectively, our results suggest that loss of Prdx6 caused by dysregulation of ARE/Nrf2 can be attenuated through a SFN, to combat diseases associated with aging.

## Introduction

A prominent feature of biological aging is a progressive decline in antioxidant defense mechanisms, which are crucial to protecting cells and tissues from many oxidative, chemical and pathological stresses^1–6^. The decline in antioxidant defenses gives rise to age-related diseases that result from increased levels of reactive oxygen species (ROS)-driven stress^1,3,5–11^. Exposure of mammalian cells to environmental stressors like UVB generally initiates antioxidant transcriptional responses. These responses involve the coordinated upregulation of antioxidant genes, glutathione S-transferase (GST*π*), catalase (Cat), glutathione-peroxidase (GPxs), hemeoxygenase1 (HO-1) and peroxiredoxins (Prdxs), to lessen the oxidative load and restore cellular homeostasis. The antioxidant proteins are present in lens, contributing to maintenance of lenticular physiology^1^. The transcriptional regulation of these cytoprotective genes is tightly controlled by *cis*-acting elements, known as antioxidant response elements (ARE), present in the enhancer region^12^. Several reports show that activity of Nrf2 declines with age, but the causes of the decline are not well understood. There is evidence that Nrf2 loses its DNA binding to ARE, and that loss of Nrf2/ARE binding may be reversible by an Nrf2 agonist such as α-lipoic acid^13^. This suggests that Nrf2-mediated survival pathways are responsive and correctable, and that correction may require phytochemicals that are efficacious in regulating Nrf2/ARE pathway(s) and deliverable. One such phytochemical is the naturally occurring compound Sulforaphane (SFN)^14^.

Transcription factor Nrf2 is a major transactivator of cytoprotective genes in response to oxidative stress and xenobiotic electrophiles. It acts by binding to ARE present in gene promoter^15–17^. Activation of the Nrf2 pathway maintains redox homeostasis by removing ROS. Under physiological conditions, Nrf2 is regulated by cytoplasmic Keap1, an adopter protein for CULLI3-based ubiquitin E3 ligase that continuously ubiquitinates Nrf2 for proteasomal degradation. Upon oxidative stress/exposure to electrophiles, Keap1 is inactivated due to electrophile binding. The inactivation leads to dislodging of Nrf2 from Keap1, and allows Nrf2 to escape degradation^16^. Nrf2 then translocalizes into the nucleus, where it activates target detoxifying and antioxidant genes^18^. Recently, several reports have shown that SFN is a potent cytoprotective with diversified functions, and that its protective ability depends upon concentration and cellular background^19,20^. Cancer cells may respond differently to SFN. In certain tumor cells, Keap1 is mutated, leading to constitutive activation of Nrf2^21^. Loss of Nrf2 expression in cancer cells increases oxidative damage that can lead to a reduction in tumorigenesis^22–24^. Nonetheless, SFN is not a direct antioxidant, but acts by regulating the Nrf2 pathway^25,26^. Thus, SFN can produce different responses in different cell types. Because of its electrophilic property, SFN induces Nrf2 translocalization and accumulation in nucleus. Moreover, SFN may also mediate the phosphorylation of Nrf2 by activating various kinases, MAP, PKC and Akt, where it alters nuclear and cytoplasmic trafficking and Nrf2 integrity and stability^27–30^.

Prdx6 belongs to a new family of non-seleno peroxidases that have GSH peroxidase and acidic calcium-independent PLA_2_ (PhospholipaseA_2_) activities^9,31,32^. In stressed conditions, Nrf2 activates transcription of Prdx6^1,9,33^. Members of the Prdx family are divided into two categories based on the number of cysteine residues: 1-Cys and 2-Cys Prdxs. The reaction mechanism of 1-Cys Prdx (Prdx6) is different from that of the 2-Cys Prdxs, because the 1-Cys Prdx lacks a COOH-terminal Cys residue^34^. The unique capacity of Prdx6 to regulate signaling and maintain phospholipid turnover distinguishes Prdx6 from the other five members of the Prdx family (Prdx1 to 5). Prdx6 is widely expressed, and high levels have been found in lung, eye lens, keratinocytes, skin and brain^1,9,35–37^. Its reduced expression can lead to cell death, tissue degeneration and development and progression of several diseases including oxidative-induced cataractogenesis^8,38^, psoriasis^39^, and atherosclerosis^40^. ROS within cells is generated by different compartments (organelles), such as mitochondria, endoplasmic reticulum, plasma membrane and so on, which can be critical determinants of either deleterious or redox signaling. For survival signaling, antioxidant molecules should be localized into these compartments to establish redox signaling. In this regard, Prdx6 is localized in these compartments^41,42^. Aside from this, we have shown that Prdx6 is localized in the lens as well as in lens fibers; however, Prdx6 was dramatically reduced with age, and was present only in the cortical fibers and the germinative zone of mouse or rat lenses^1,8^. Interestingly, a dramatic correlation has been found between higher nuclear cataract scores and lower expression of Prdx6 in patients^8,33^. In the current study we found a significant loss of Nrf2 in aging, which was connected to suppression of Prdx6 with increased levels of ROS. Because ARE-type *cis*-acting sequences exist in the regulatory region of Prdx6 promoter (−357/−349), we envisaged that a potential loss of Prdx6 might be related to Nrf2 dysregulation in aging.

Using eye lens and lens epithelial cells (LECs), one of the best biological systems for study of molecular mechanisms of age-related diseases, we found that an age-associated decline in Prdx6 expression was linked to the loss of Nrf2. This dysregulation of Nrf2 was reflected in its reduced expression and DNA-binding activity to ARE. Importantly, we showed that dysregulation of Nrf2/ARE pathways was responsive to SFN, and SFN was able to restore Nrf2 transactivation potential, leading to expression of Prdx6 and cytoprotection against UVB-induced injury. Testing other antioxidants, GST*π* and Cat, we observed that SFN also induced their expression, suggesting that SFN can repair and regulate basal Nrf2/ARE signaling in lens. Thus, we propose that SFN mediates activation of this molecular switch, and that restoration of the Nrf2/Prdx6 pathway provides a proof of concept that SFN can be considered as a therapeutic chemo-protectant to repair and reverse age-related diseases, such as cataractogenesis.

## Results

### Age-related increased oxidative load in LECs was linked to progressive decline in Nrf2, Cat and Prdx6 expression

**Figure 1:**
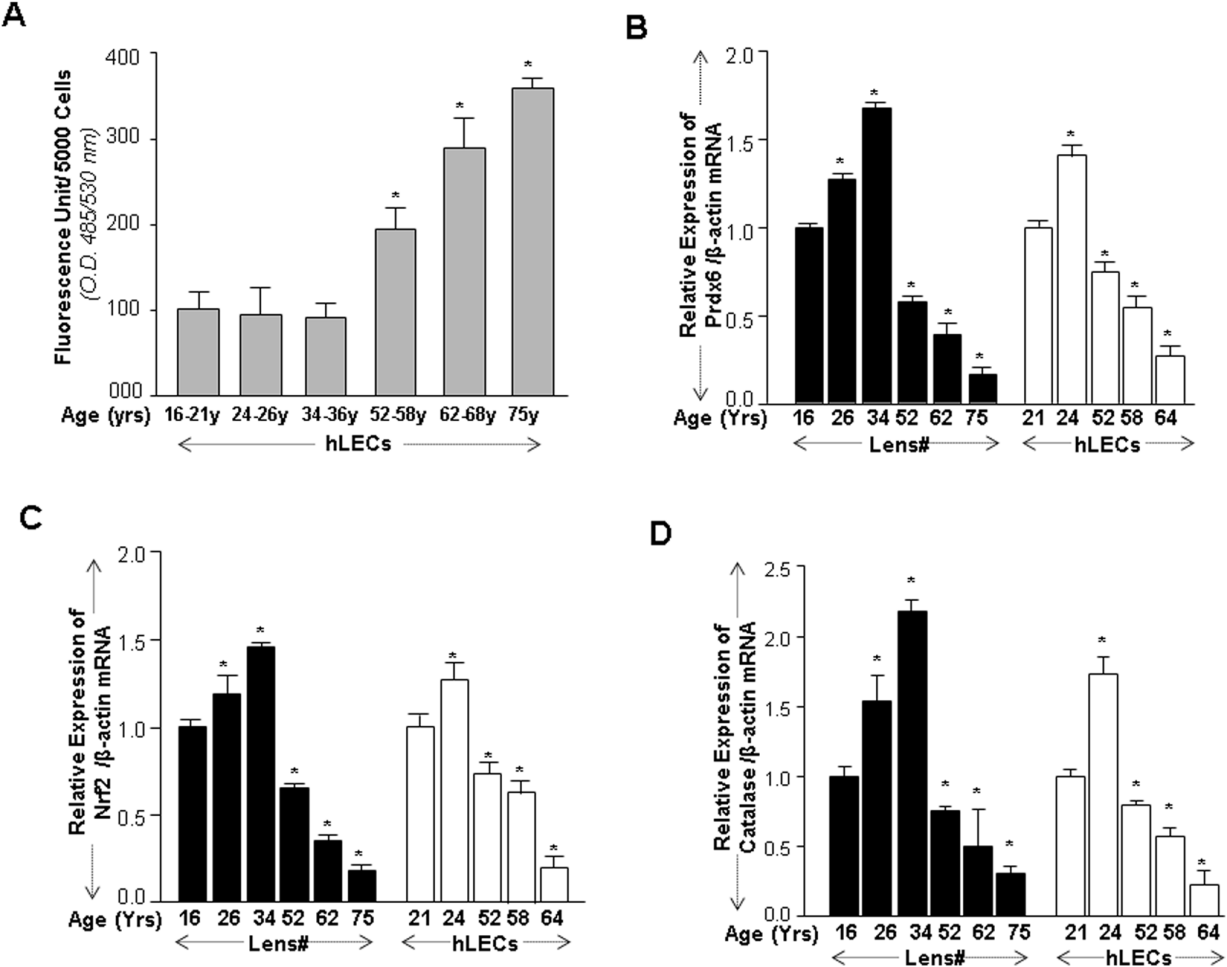
Aging/aged hLECs displayed increased accumulation of ROS, which was associated with progressive decline in Prdx6, Cat and Nrf2 expression. (**A**) Excessive accumulation of ROS in aging/aged hLECs. Primary hLECs isolated from lenses of different ages were divided into six groups: 16–21 y (n = 6); 24–26 y (n = 6); 34–36 y (n = 4); 52–58 y (n = 6); 62–68 y (n = 12); 75 y (n = 4). Cells were cultured in 96 well plate (5000/well), and ROS were quantified using H2-DCF-DA dye assay as shown. Data represent the mean ± S.D. of two independent experiments. 16–21 y vs 24–26 y, 34–36 y, 52–58 y, 62–68 y and 75 y (aging samples); *p < 0.001. (**B**–**D**) Aging/aged hLECs showing a significant loss of Prdx6, Cat and Nrf2. Total RNA was isolated from hLECs and human lenses of different ages as indicated and was processed for real-time PCR analysis. ^#^LECs directly detached from lenses and were used for assays to avoid cell culture effects. The data represent the mean ± S.D. from three independent experiments. *p* values were determined for younger vs aging samples. *p < 0.001.

To identify age-related changes in ROS production and the connection between expression of Prdx6 and its regulator Nrf2, an antioxidant defense pathway, we monitored the intracellular redox-state of primary hLECs of different ages cultured in 96 well plate by using H2-DCF-DA dye^8,43^. Quantification by staining with H2-DCFH-DA dye revealed an age-dependent progressive increase in ROS levels (Fig. 1A), and a higher abundance of ROS was noted in aged hLECs (Fig. 1A, 52 y onward)^44^. Figure 1A reflects the ROS levels in pooled samples of LECs derived from lenses of different age groups as described in the Methods and figures Legends section. This result prompted us to manipulate experiments to maximize the limited supply of primary hLECs. To discern if the apparent increase in ROS levels during aging was due to loss of Nrf2/Prdx6, mRNA from the same group of lenses/hLECs of different ages was isolated and was quantified with qPCR. Data analysis revealed that lens/hLECs mRNA expression of Prdx6, Cat and Nrf2 declined with aging, and this loss was more significant in aged cells (Fig. 1B–D). As expected, we found a significant inverse correlation between expression of Nrf2/Prdx6 and increased ROS levels during aging.

### Binding of Nrf2 to ARE in Prdx6 was functionally dysregulated with aging

**Figure 2:**
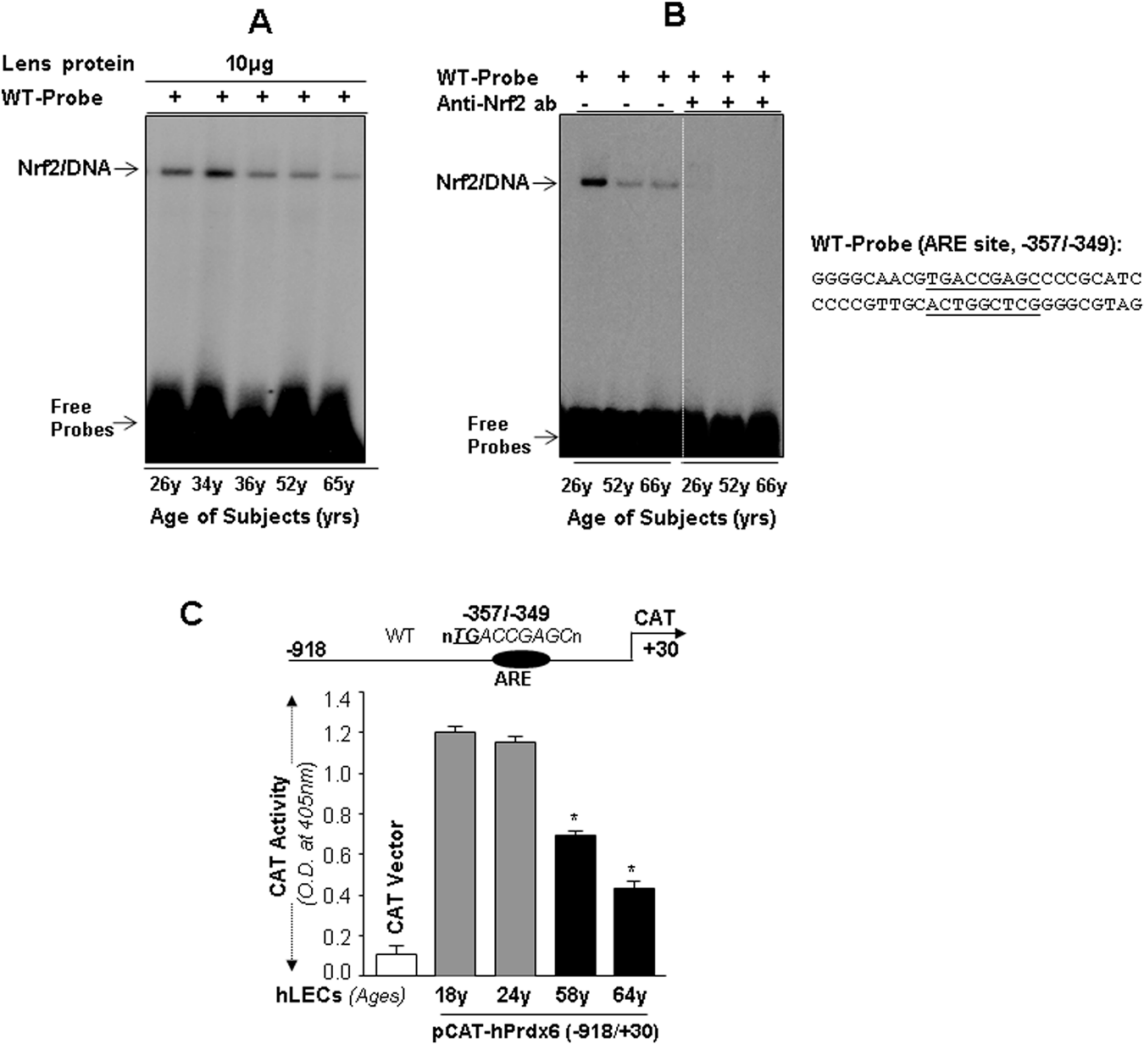
Aging hLECs displayed a significant loss in Nrf2 binding to ARE and in transactivating Prdx6 promoter activity. (**A**) Gel-shift with nuclear extract from lenses of variable ages shows age-related loss of Nrf2 binding to ARE in Prdx6 promoter. Nuclear fraction directly isolated from hLECs of different ages containing equal amounts of protein were incubated with ^32^p-labeled wild-type ARE probe from Prdx6 promoter and processed for gel-shift assay. An apparent age-related reduction in Nrf2/ARE binding was observed (A, Nrf2/DNA). (**B**) Nrf2-specific antibody depletion assay revealing depletion of Nrf2/ARE complex, demonstrating specificity of Nrf2 binding to ARE probe. Equal amounts of nuclear protein were incubated with antibody specific to Nrf2 to deplete Nrf2. No Nrf2/DNA band was detected with Nrf2-depleted extracts (B, lanes: left panel; 26 y, 52 y and 66 y vs right panel; 26 y, 52 y and 66 y). Wild-type probe, underlined bases denote ARE sequences (−357/−349) present in Prdx6 regulatory region. (**C**) Age-related transcriptional activity of Prdx6 promoter in primary hLECs of variable ages. Top panel, diagrammatic sketch showing the 5′- constructs of human Prdx6 promoter ranging from −918/+30 bps linked to CAT reporter gene. Lower panel, histogram showing CAT activity of Prdx6 promoter and empty CAT vector. Cells were transiently transfected with Prdx6 promoter plasmid along with pGFP-vector plasmid. 48 h later, CAT activity was monitored (Methods section). Transfection efficiency was normalized with GFP O.D. recorded at Ex485/Em530nm. Data represent the mean ± S.D. from two independent experiments. Younger age (18 y and 24 y) vs aging sample; *p < 0.001.

We next examined whether the age-related decline of Prdx6 mRNA is associated with loss of Nrf2 binding to ARE present in the Prdx6 promoter. We carried out gel-shift assay with nuclear fraction of hLECs directly detached from lens (to avoid cell culture effects) selected from the same age group of lenses that were used in the previous experiment (Fig. 1A). As shown in Fig. 2A, we found progressively reduced Nrf2 binding to^32^ p-oligonucleotide containing ARE during aging. The lowest level of binding was displayed with nuclear fraction from older hLECs (Fig. 2A). Furthermore, Nrf2 depletion experiment revealed reduced or no binding to probe in gel-shift experiments (Fig. 2B, right panel; ages 26 y, 52 y and 66 y) compared to the control (Fig. 2B, left panel; 26 y, 52 y and 66 y), demonstrating that Nrf2 specifically bound to probe and formed complex (Nrf2/DNA). Next we tested the functionality of Nrf2 binding by using transactivation assay. Cultured primary hLECs of different ages were transfected with Prdx6 promoter containing ARE sequences (Fig. 2C, top panel). We observed a significant decline in Prdx6 promoter activity in aging cells (Fig. 2C, gray vs black bar), which was directly related to the decline in Nrf2 binding to ARE. Collectively, our results demonstrated the functional loss of Nrf2’s activity in aging.

### Sulforaphane induced Nrf2-dependent ARE-antioxidant gene transcripts in LECs

**Figure 3:**
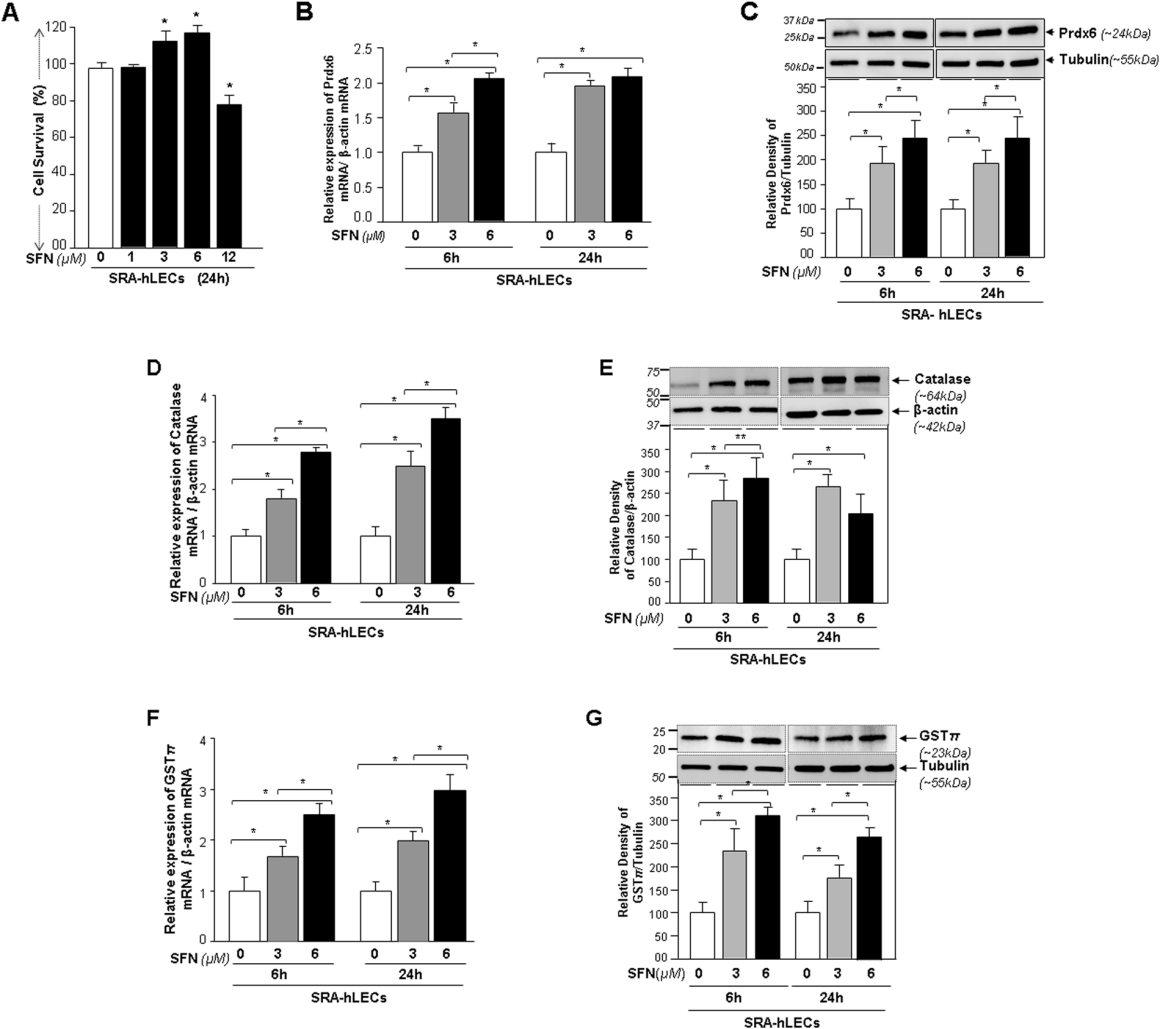
SFN enhanced expression of antioxidants Prdx6, Cat and Phase II protein GST*π* mRNA and protein in dose-dependent manner in SRA-hLECs. (**A**) Viability assay showing the concentration-dependent effects of SFN on survival of SRA-hLECs. Cultured SRA-hLECs were treated with different concentrations of SFN to determine a nontoxic concentration of SFN using MTS assay. DMSO vs SFN treated; *p < 0.001. (**B** and **C**) SFN significantly enhanced Prdx6 mRNA and protein expression. Cells were treated with DMSO vehicle or different concentrations of SFN for 6 h and 24 h. mRNA and protein were extracted, and subjected to real-time PCR and immunoblotting using probes specific to Prdx6. SFN produced a concentration-dependent increased pattern of Prdx6 mRNA (**B**) and protein (**C**) expression. (**D** and **E**) As noted in B and C, mRNA and cellular extract isolated from cells treated or untreated with SFN were submitted to real time-PCR (D) and immunoblot (E) analyses using primers and antibody specific to Cat, respectively. (**F** and **G**) SFN also significantly augmented levels of GST*π*, a phase II enzyme. In parallel experiments the expression level of GST*π* was examined in SFN-treated cells by real-time PCR (**F**) and Western analysis (**G**). (**C,E** and **G**); Upper panel shows a representative of immunoblot; lower panel; protein bands were quantified using a densitometer, and levels were normalized to corresponding β-actin levels with values presented as histograms. (**B–G**), Data represent means ± S.D. of three independent experiments. Open vs gray and black bars; gray vs black bar; *p < 0.001, **p < 0.05.

**Figure 4:**
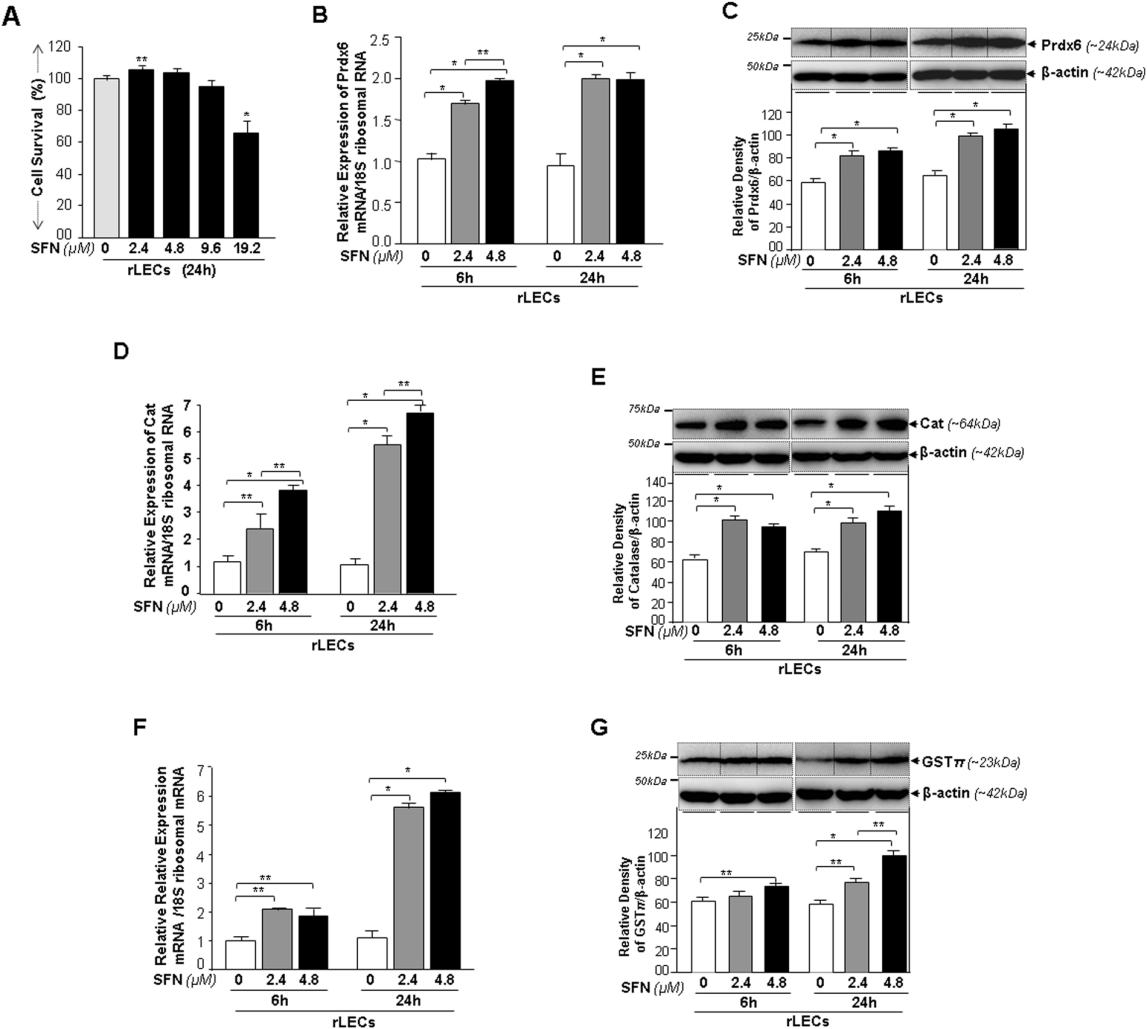
Rat LECs treated with SFN displayed increased levels of antioxidant genes/proteins, Prdx6, Cat, and phase II protein GST*π*. **(A)** Determination of noncytotoxic concentration of SFN in primary culture of rLECs. Primary cultures of rLECs were treated with different concentrations of SFN as indicated for 24 h. Cells were subjected to MTS assay to measure viability. Histogram reflects values; nontoxic concentrations were 2.4 μM and 4.8 μM. DMSO vs SFN treated samples; *p < 0.001, **p < 0.05. (**B** and **C**) rLECs treated with SFN showed enhanced expression of Prdx6 mRNA and protein. Cells were treated with 2.4 μM or 4.8 μM of SFN or DMSO as indicated. Total RNA and protein were isolated. Real-time PCR and Western analysis with Prdx6 specific probes revealed a concentration-dependent increased expression of Prdx6 mRNA (**B**) and protein (**C**). (**D** and **E**) Expression assays showing SFN enhanced expression of Cat in rLECs. Experiments and parameters were similar to those noted above (**B** and **C**). RNA and protein extract were processed for real-time PCR and Western analyses using Cat specific primers and antibody, respectively. (**F** and **G**) SFN-treated rLECs displayed significantly increased levels of GST*π* mRNA and protein in time- and concentration-dependent fashion. mRNA and cellular extracts were isolated from SFN-treated primary rLECs, and were processed for real-time PCR (**F**) and Western analysis (**G**) assays. (**C,E** and **G**); Upper panel, representative Immunoblot. Lower panel, densitometric analysis of protein band level; levels were normalized to corresponding β-actin levels and values are presented as histograms (C and G, dotted line shows marking of boundary of bands). (**B–G)**, Data represent means ± S.D. of three independent experiments. Open vs gray and black bars; gray vs black bar; *p < 0.001, **p < 0.05.

Based on the decline in Nrf2′s expression and DNA binding ability (Figs 1 and 2), we sought to determine whether SFN would stimulate basal levels of antioxidant gene expression in LECs. Because using primary hLECs was cumbersome due to their limited availability, we utilized the SRA-hLECs and, to generalize our findings, we included primary rLECs as a model system. We first determined an effective noncytotoxic concentration of SFN as indicated in Figs 3A and 4A. Cell growth assessed at 24 h of treatment showed that concentrations of 3 µM and 6 µM and 2.4 µM and 4.8 μM had better effects on SRA-hLECs and rLECs growth, respectively. Thus, these doses were used throughout the study unless otherwise stated.

To examine the efficacy of SFN in inducing expression of antioxidants Prdx6, Cat and phase 2 detoxifying enzyme GST*π* from their basal expression in LECs, SRA-hLECs treated with SFN for 6 h and 24 h were processed for qPCR. Basal transcription of these genes was dramatically increased in SFN-treated cells, as evidenced by increased mRNA levels (Fig. 3B,D,F; Open vs gray and black bars; gray vs black bar). In another set of SFN-treated SRA-hLECs, cellular extracts immunoblotted with anti-Prdx6, anti-Cat and anti-GST*π* antibodies revealed significantly increased expression of all three proteins, The maximum expression level was detected at 6 µM of SFN concentration (Fig. 3C,E,G; Black bars), consistent with increased expression of mRNA (Fig. 3B,D,F).

Antioxidant response can differ in cell types of different genetic backgrounds. Thus, we next examined whether the results obtained in SRA-hLECs were reproducible in primary rLECs. We found that rLECs treated with SFN (2.4 µM and 4.8 µM) for 6 h and 24 h had similar increased expression patterns of transcripts of all three molecules (Fig. 4B,D,F) as observed with SRA-hLECs. Next we examined the levels of Prdx6, Cat and GST*π* protein in rLECs treated with SFN. Immunoblot analysis with their corresponding specific antibodies showed increased protein expression in SFN-treated cells (Fig. 4C,E,G). These data demonstrate that SFN activated the genes expression by enhancing their transcription in both SRA-hLECs and rLECs.

### SFN activated Nrf2 transcription and reinforced its translocalization into nucleus

**Figure 5:**
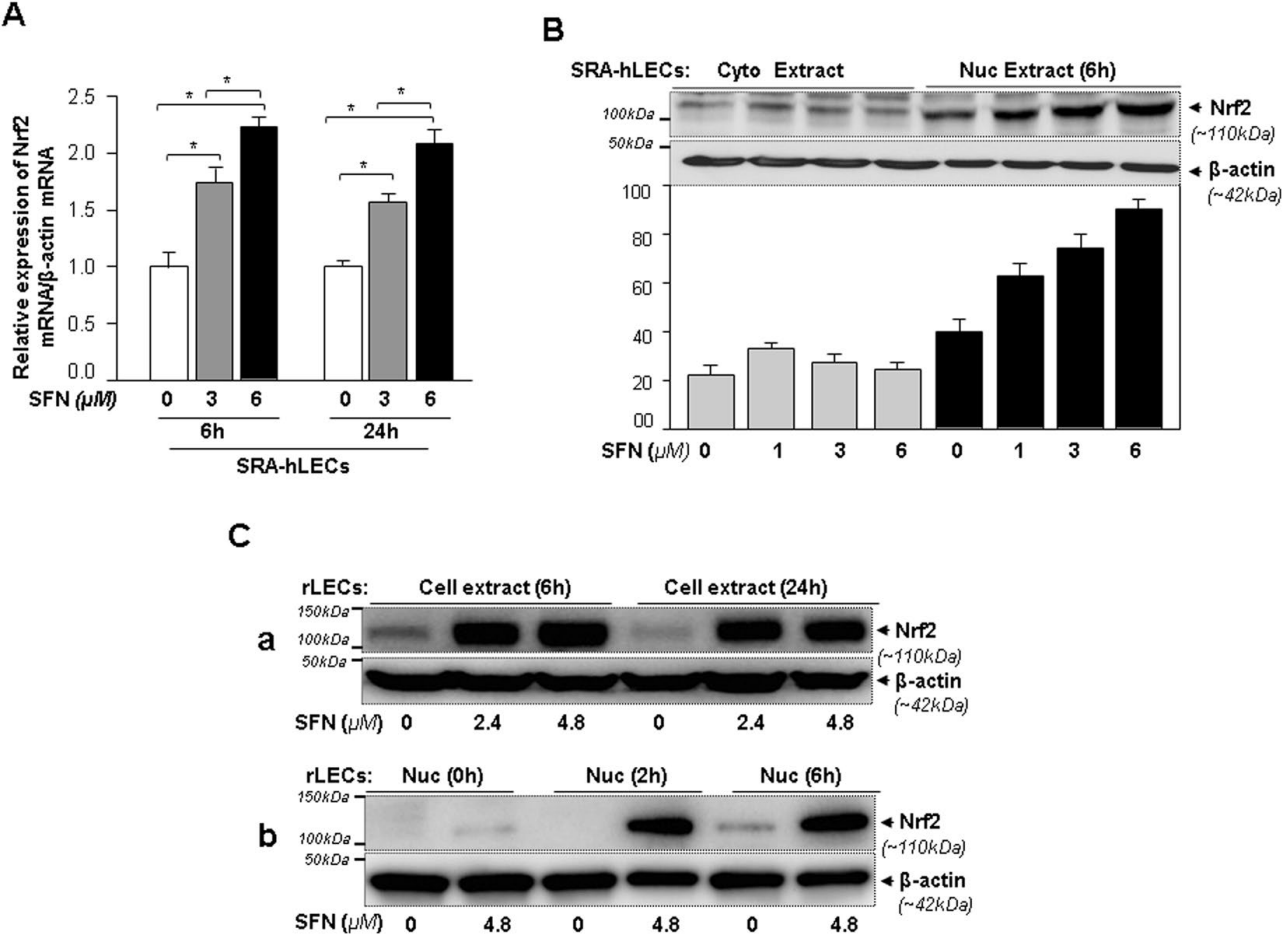
SFN induced Nrf2 expression and enhanced nuclear accumulation in both SRA-hLECs and rLECs. (**A**) Effect of SFN concentration(s) on expression of Nrf2 mRNA in SRA-hLECs. Total RNA was isolated and real-time PCR was performed using specific primers. mRNA expression of Nrf2 was adjusted/normalized to the mRNA copies of β-actin. Histogram represents mean ± S.D. obtained from three independent experiments. Open vs gray and black bars; gray vs black bar; *p < 0.001. (**B**) SFN-mediated induction of Nrf2 expression and nuclear localization. Cultured SRA-hLECs were treated with different concentrations of SFN for 6 h. Cytosol and nuclear extract were immunoblotted with anti-Nrf2 antibody. β-actin was used as loading control. Upper panel, An apparent increased nuclear translocalization of Nrf2 was observed. Lower panel, Histogram showing relative density of protein bands (Nrf2/β-actin). (**C**) Nrf2 activation by SFN accompanied accumulation of Nrf2 in whole cell lysates and led to nuclear accumulation in time- and concentration-dependent fashion in rLECs. Primary culture of rLECs treated with different concentrations of SFN were processed for extraction of total cell extract as well as cytosolic and nuclear fractions at predefined time intervals as indicated. Cellular extract (**C**, a) or nuclear fraction (**C**, b) containing equal amounts of protein were immunoblotted with anti-Nrf2 antibody. β-actin was used as loading control. A significant accumulation of Nrf2 in nucleus was observed when examined at 2 h and onwards compared to basal levels (untreated control).

To establish the molecular mechanism of the Nrf2 activation in LECs, we examined the time-and dose-dependent effect of SFN regulation of Nrf2 expression by using the same concentrations of SFN and durations of treatment which had been found effective in activating antioxidant genes (Figs 3 and 4). SRA-hLECs were treated with SFN as shown in Fig. 5A and qPCR was conducted. The mRNA levels of Nrf2 increased with SFN treatment (Fig. 5A), emphasizing that Nrf2 can be an activator of its own transcription as previously reported^45^. Next we examined the Nrf2 protein level in cytosolic and nuclear extracts of SRA-hLECs treated with different concentrations of SFN for 6 h (the time at which mRNA was at its peak). Immunoblot data using anti-Nrf2 antibody revealed that Nrf2 migrated at approximately 110 kDa-in SDS-PAGE, which was enriched in nuclear extract of SRA-hLECs, and maximum accumulation occurred at 6 μM of SFN concentration as shown in Fig. 5B. Conversely, cytosolic extract had a residual minimal amount of Nrf2 protein. However, Western analysis revealed more than two faint bands. A knockdown experiment (shNrf2) coupled with immunoblotting with anti-Nrf2 antibody (Supplementary Fig. S1A) revealed that the band with strong density shown in Fig. 5B was specific to Nrf2. We also observed the presence of Nrf2 in nuclear fraction of untreated control SRA-hLECs. This argues that a low level of Nrf2 in nuclear fraction of LECs may be necessary for basal expression of protective genes in favor of maintaining cellular activity.

Next, to discern activation of the antioxidant response after SFN treatment, we examined cellular and subcellular changes in Nrf2 disposition in rLECs. In untreated control cells, Nrf2 was present at very low levels in whole cell extract and was barely detectable, demonstrating that ongoing proteasomal degradation machinery was active during normal physiological conditions (Fig. 5C, panel a and b)^18,46^. We observed that cells treated with SFN showed Nrf2 accumulation in total cell extracts within 6 h at each concentration (Fig. 5C, panel; a), which is consistent with SFN-mediated inactivation of Keap1 as noted in Introduction section. Because both concentrations of SFN were effective, we chose only the higher concentration, 4.8 μM, to treat cells for shorter time (2 h) to examine how quickly Nrf2 translocated/accumulated into nucleus. Nuclear fraction isolated from SFN-treated and -untreated rLECs were immunoblotted as shown in Fig. 5C, panel; b. A significant accumulation of Nrf2 was detected in nuclear fraction of SFN-treated rLECs when observed at 2 h (Fig. 5C, panel; b), suggesting this initial lag period may be necessary for translational synthesis of new Nrf2 protein^47^, and also that the time period of within 2 h may represent the time critical for nuclear translocation and ARE-mediated gene transcription. As a whole, the data indicated increased cellular abundance of Nrf2 but nuclear accumulation, a basic phenomenon occurring in SRA-hLECs and rLECs during SFN induction of Nrf2 as described previously for other cells^26,48^.

### Upregulation of antioxidant genes in SRA-hLECs/rLECs was largely derived from SFN-induced augmented Nrf2 binding to ARE

**Figure 6:**
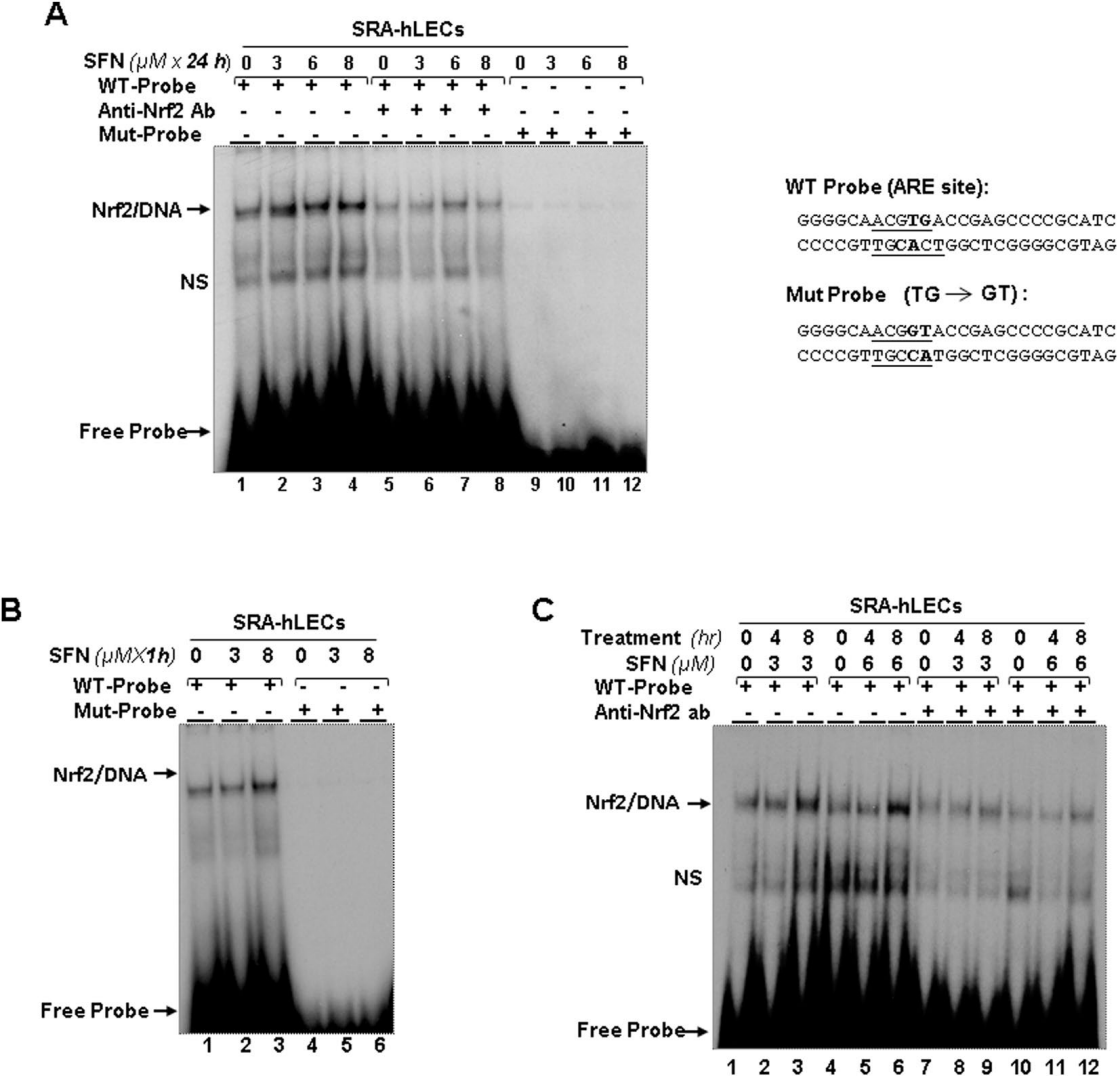
SFN enhanced physical and functional binding of Nrf2 to ARE present in the regulatory region of human Prdx6 promoter in SRA-hLECs. (**A**) Gel-shift and antibody depletion assay showed SFN enhanced Nrf2 binding to oligo probes containing ARE derived from Prdx6 promoter in concentration-dependent fashion. Nuclear fraction extracted from SRA-hLECs was incubated with ^32^p radiolabeled wild-type or mutant probes containing ARE sites. SFN concentration-dependent binding activity of Nrf2 to ARE (Nrf2/DNA; lanes 1 vs 2, 3 and 4) compared to mutant probe (lanes 9, 10, 11 and 12). Antibody depletion assay showed disruption of Nrf2/DNA complex (lanes 5, 6, 7 and 8), suggesting that Nrf2 in nuclear extract selectively bound to ARE. (However, antibody did not entirely deplete Nrf2 in nuclear faction of SRA-hLECs, so some residual interaction can be seen in all lanes.) (**B**) SFN rapidly stimulated Nrf2 binding activity to ARE present in Prdx6 human promoter. SRA-hLECs were cultured in the presence of DMSO (control vehicle) or with different concentrations of SFN for 1 h. Nuclear fractions were isolated and processed for gel-shift assay. A strong Nrf2/DNA complex was formed with SRA-hLECs treated with 8 μM of SFN for 1 h (B, lane 1 vs 2 vs 3). In contrast, mutant probe did not act similarly, validating that the Nrf2/DNA complex on gel-shift was specific. (**C**) Gel-shift and antibody depletion assay showed SFN amelioration of Nrf2 binding activity to ARE in the Prdx6 promoter in concentration- and time-dependent fashion. SRA-hLECs were treated with different concentrations of SFN for different time periods. Nuclear extracts containing equal amounts of proteins were incubated with radiolabeled ARE probe. A relative modulation in Nrf2/DNA complex intensity was observed, and was related to concentration and time of exposure as shown in a representative figure (C, lane 1 vs 2 and 3; Lane 2 vs 3 and lane 4 vs 5 and 6; lane 5 vs 6). In contrast, antibody depletion assay showed reduced band intensity or ablation of DNA/Nrf2 complex (C, lanes 7, 8, 9, 10, 11 and 12). Bold bases represent mutation sites; mutated base(s) as shown and underlined denote core ARE sequences in Prdx6 promoter. NS denotes nonspecific band.

To determine whether SFN activation of Prdx6 transcription in SRA-hLECs resulted from a gain in DNA binding activity of Nrf2 to ARE, nuclear fraction from SFN treated SRA-hLECs (0, 3 μM, 6 μM, 8 μM) for 24 h were tested on gel-shift assay. We synthesized the oligonucleotides derived from Prdx6 promoter containing ARE (^−357^n***TG***ACCGAGCn^−349^) and its mutant containing GT binding sites (Fig. 6). Nuclear fraction from SFN-treated cells showed enhanced binding to ARE and formed a shifted complex (Fig. 6A, Nrf2/DNA; lanes: 2, 3 and 4) compared to control (Fig. 6A, lane1). The increase of binding was related to increased concentrations of SFN (Fig. 6). The shifted Nrf2/DNA complex that appeared in lanes was diminished when the Nrf2-depleted nuclear extract was used for binding assay (Fig. 6A, lanes, 5 to 8). Nonetheless, there was mild interaction between Nrf2-depleted nuclear extract to probe, which may have occurred because antibody concentration was not optimal for absolute depletion of Nrf2 (Lane 5 vs 6, 7 and 8). In addition, nuclear extract did not interact with the mutant probe (Fig. 6A, Mut probe; lanes, 9 to 12), verifying the specificity of ARE/Nrf2 binding.

### SFN enhanced interaction of Nrf2/ARE in SRA-hLECs in a time- and concentration-dependent manner

Given the apparent inductive response of antioxidant genes (Figs 3 and 4) to SFN, we sought to determine how effectively SFN activated Nrf2/ARE interaction in SRA-hLECs. Using gel-shift assay, we determined the SFN-induced kinetics of Nrf2/ARE interaction. We treated cells with two concentrations, 3 μM and 8 μM of SFN, based upon our previous finding. Gel-shift assay with the same ARE probe and its mutant as shown in Fig. 6A demonstrated that the higher concentration of SFN enhanced Nrf2 binding and formed Nrf2/DNA complex (Fig. 6B, lane 3) compared to the lower concentration (Fig. 6B, lanes, 1 and 2; respectively). In contrast, with the mutant probe, nuclear fraction of SRA-hLECs did not show the Nrf2/ARE complex (lanes 4, 5 and 6), indicating specificity.

Because a 3 μM concentration of SFN for 1 h did not affect Nrf2/ARE interaction significantly, we examined the effect of duration of SFN treatment on the interaction. SRA-hLECs treated with 3 μM and 6 μM of SFN for 4 h and 8 h were processed for gel-shift assay. Figure 6C shows a time-dependent increase of Nrf2/ARE binding (Fig. 6C). A closer observation of Nrf2/ARE complex revealed that maximum binding occurred in nuclear fraction of SRA-hLECs treated with either concentration for 8 h (Fig. 6C, lanes 1 and 2 vs 3 and lanes 4 and 5 vs 6). No or significantly reduced interaction was observed in Nrf2-depletion assay (lanes 7 to 12). As whole, our results demonstrate the time- and concentration-dependent effect of SFN on ARE-mediated gene expression.

### *In vivo* DNA protein binding assay revealed that SFN enhanced Nrf2 enrichment at ARE sequences present in the Prdx6 promoter

**Figure 7:**
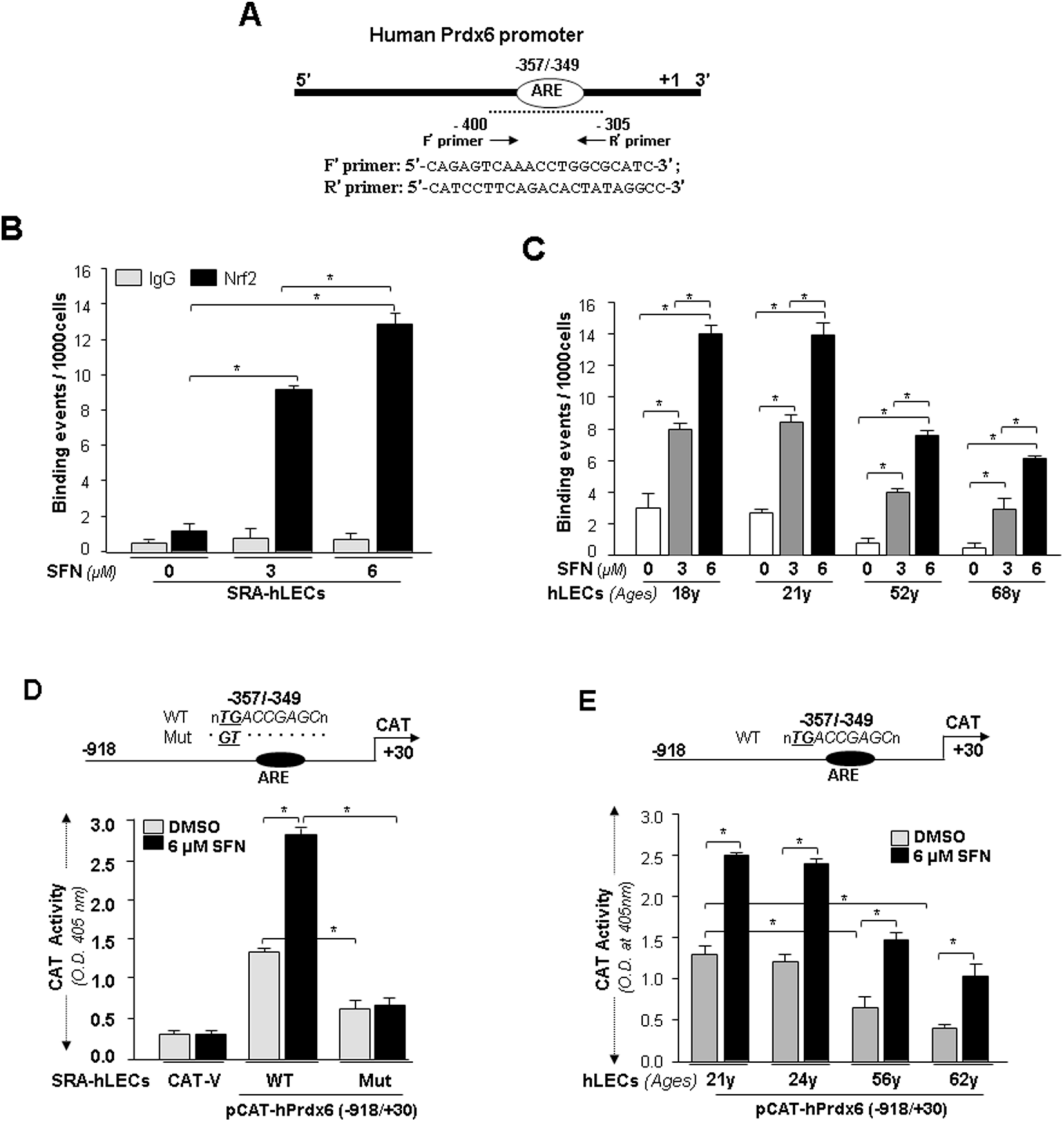
*In vivo* DNA binding assay revealed that SFN reinforced binding activity of Nrf2 in SRA-hLECs and aging/aged primary hLECs. (**A**) Schematic representation of the regulatory region of proximal promoter of human Prdx6 gene-containing ARE binding sites showing primer location and sequences used in ChIP assay. **(B)** SFN induced increase in DNA binding activity of Nrf2 to Prdx6 gene promoter containing ARE site in SRA-hLECs. ChIP experiment was carried out by using ChIP-IT® Express and ChIP-IT® qPCR analysis Kit. Chromatin samples prepared from SRA-hLECs treated with varying concentrations (0, 3 µM and 6 µM) of SFN for 24 h were subjected to ChIP assay with a ChIP grade antibody, anti-Nrf2 (black bars) and control IgG (gray bars). The DNA fragments were used as templates for qPCR by using primers designed to amplify −400 to −305 region of the human Prdx6 promoter bearing Nrf2/ARE sites as shown. Histogram shows the amplified DNA band visualized with real-time PCR analysis. DMSO (0) vs 3 µM and 6 µM SFN and 3 µM vs 6 µM SFN treatment; *p < 0.001. (**C**) SFN reinforced the enrichment of Nrf2 to its responsive element, ARE, present in Prdx6 gene promoter in aging/aged primary hLECs. ChIP assay was conducted using anti-Nrf2 antibody. Immunoprecipitated DNA fragments were purified and processed for qPCR analysis using primers indicated above and in the Methods section, but in primary hLECs of variable ages treated with different concentrations (0, 3 µM, 6 μM) of SFN. Histograms represent the concentration dependence of SFN-induced enrichment of Nrf2 at ARE sites in Prdx6 gene promoter. Open vs gray and black bars and gray vs black bar; *p < 0.001. Data revealed a significant augmentation of Nrf2 binding to ARE by SFN in all ages of LECs, but in aged cells there was a loss in Nrf2 binding to ARE. (**D**) SFN failed to activate mutant Prdx6 promoter disrupted at Nrf2/ARE site. Upper panel, diagram of 5′-regulatory region of Prdx6 promoter spanning from −918/+30 bp containing ARE site and its mutant plasmid linked to CAT reporter gene used for CAT activity. Lower panel, CAT activity of the wild-type (WT) Prdx6 promoter and its mutant (Mut) at ARE site and empty CAT vector in SRA-hLECs treated with SFN or DMSO (control). Wild-type or its mutant Prdx6 promoter construct along with pGFP-Vector were cotransfected into SRA-hLECs and CAT activity was measured. CAT activity (lower panel) was normalized to GFP readings (O.D.). Histogram represents the mean ± S.D. obtained from three independent experiments. WT vs Mut and DMSO vs SFN treated samples; *p < 0.001. (**E**) SFN reinforced Prdx6 transcription in aging/aged primary human LECs. As described above, hLECs of variable ages were transfected with the same wild-type Prdx6 promoter ARE site (upper panel). Lower panel, relative CAT activity of the wild-type promoter in SFN-treated aging/aged hLECs. All data are presented as mean ± S.D. values derived from three independent experiments. DMSO vs SFN treated samples; younger (21 y old) vs aging samples; *p < 0.001.

Careful analysis of *in vitro* data on SFN-induced Nrf2/DNA interaction showed that Nrf2 exclusively bound to ARE (Fig. 7A). Next, to determine if increased activation of Nrf2 occurred via a direct mechanism *in vivo*, we employed chromatin immunoprecipitation (ChIP) assay to measure the occupancy of Nrf2 on ARE of hPrdx6 gene promoter. SRA-hLECs treated with SFN (0 μM, 3 μM and 6 μM) for 24 h were processed for ChIP assay with anti Nrf2 antibody (Fig. 7A) as described in the Methods section^49^. Figure 7B shows that the Prdx6 promoter containing ARE sequences was occupied by Nrf2, and increased enrichment of Nrf2 to the sequences was SFN concentration-dependent. No amplicon was observed with control IgG, pointing to specificity of Nrf2 antibody. These data demonstrate that SFN enhanced Nrf2 enrichment at ARE sequences, and explain the mechanism of SFN-dependent increased Prdx6 transcription.

To test the efficacy of SFN in activating Nrf2 in aging/aged hLECs, we performed ChIP assay. Aging hLECs treated with SFN (0 μM, 3 μM and 6 μM) for 24 h were processed for ChIP assay with Prdx6 promoter as mentioned above. As shown in Fig. 7C, the enrichment of Nrf2 at ARE sequences in Prdx6 promoter was significantly increased in SFN-treated older/aged LECs in a concentration-dependent fashion (Fig. 7C, open vs gray and black bars; gray vs black bar). However, younger LECs were relatively more responsive to SFN treatment. Thus it appears that the aging hLECs retained Nrf2 activity when exposed to SFN. However, we did not perform Western analysis of SFN-treated cells to examine the nuclear or cytosolic levels of Nrf2; nonetheless ChIP experiments directly provided evidence of a concentration-dependent enrichment of Nrf2 at ARE site.

### SFN’s failure to activate mutant *Prdx6* promoter demonstrated that transactivation was largely derived from direct binding of Nrf2 to ARE in *Prdx6* promoter *in vivo*

To examine the consequences of the SFN-induced changes in Nrf2 binding to ARE on Prdx6 transcription, we transfected SRA-hLECs with WT-Prdx6 promoter-CAT construct containing ARE or its mutant (Fig. 7D, Top drawing) along with GFP plasmid. These transfectants were treated with SFN (DMSO or 6 μM) for 24 h. Transactivation assay with mutant construct showed significant inhibition in CAT activity, and SFN failed to activate it (Fig. 7D). Conversely, wild-type promoter displayed robust promotion of CAT activity in response to SFN (Fig. 7D, WT; gray vs black bar), suggesting that SFN upregulated Prdx6 transcription through ARE. However, data revealed that mutation at ARE site did not completely abolish Prdx6 promoter activity, indicating the possible involvement of other transcriptional proteins or pathways.

To examine whether SFN restored Nrf2 dysregulation of Prdx6 transcription in aging hLECs, we transfected hLECs with WT-Prdx6-CAT (Fig. 7E). SFN significantly enhanced transcriptional activity of Prdx6 from basal activity levels in all aging cells (gray vs black bar). Younger cells were more responsive than aged cells, and the response was directly related to Nrf2/ARE interaction shown in Figs 6 and 7.

### Prdx6-knockdown disclosed that SFN-treated LECs gained resistance against UVB-induced cellular insults though Prdx6

**Figure 8:**
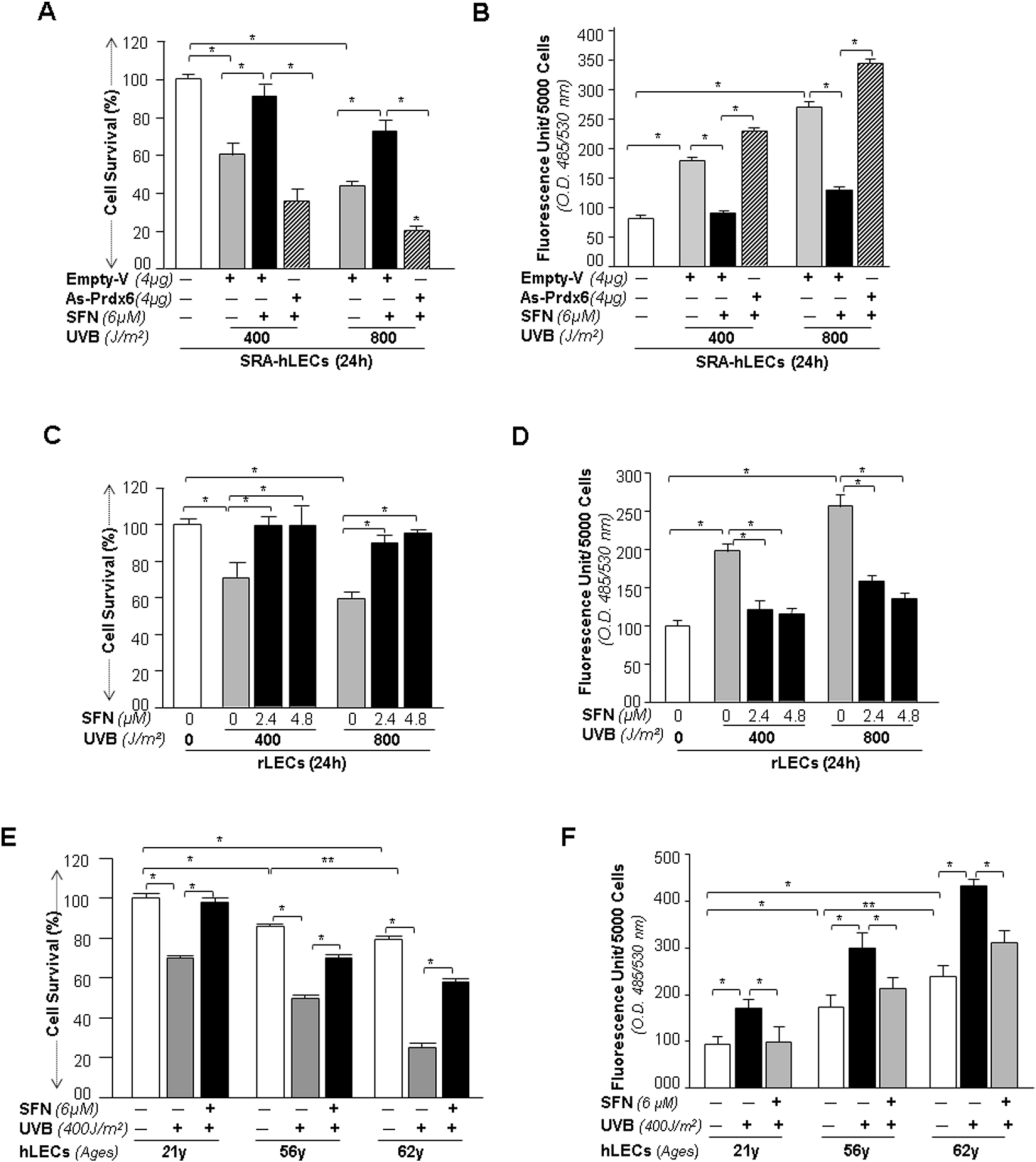
Prdx6 knockdown experiments revealed that SFN exerted its cytoprotective activity against UVB-induced cell injuries through Prdx6 regulation. **(A)** Survival experiment showing increased susceptibility of As-Prdx6 transfected cells to UVB-induced oxidative stress. SRA-hLECs were transfected with As-Prdx6 (4 μg), and the effect of As-Prdx6 was confirmed through immunoblotting with anti-Prdx6 antibody (data not shown). The transfectants were divided into different groups as shown and equal numbers of cells were cultured for assay to avoid transfection effect. Survival assay (MTS assay) showed a significant reduction in viability of As-Prdx6 (lined bar) transfectants compared to empty-vector transfectants (gray and black bars) against UVB stress. (**B**) H2-DCF-DA assay showing ROS levels after UVB stress as indicated. Result are presented as Fluorescent Unit. (**A** and **B**), Open vs gray bar, gray vs black bar and black vs lined bar; *p < 0.001. (**C** and **D**) SFN protected primary rLECs against UVB exposure. (**C)** rLECs were pretreated with 2.4 µM and 4.8 μM of SFN or DMSO (vehicle control) and then exposed to UVB stress. Effects on viability were determined after 24 h by MTS assay. (**D**) Effect of SFN on lowering ROS expression. rLECs were treated with DMSO, 2.4 µM or 4.8 μM of SFN and were exposed to UVB stress as indicated. ROS expression was quantified. All histograms are presented as the mean ± S.D. values derived from two independent experiments. C and D, open vs gray bar and gray vs black bar; *p < 0.001. (**E** and **F**) SFN rescued primary aging hLECs from UVB stress. (**E**) SFN augmented viability of aging hLECs undergoing UVB stress. Cultured hLECs of variable ages were exposed to UVB as indicated, and effects on cell viability were determined after 24 h by MTS assay. (**F**) Effect of SFN on lowering ROS expression. hLECs were treated with SFN as in (E). ROS levels were measured with H2-DCF-DA. Histogram represents the data mean ± S.D. obtained from two independent experiments. 21 y vs 56 y and 62 y, 56 y vs 62 y, Open vs gray bar and gray vs black bar; **p < 0.05, *p < 0.001.

With the goal of developing transcription-based “inductive therapy” to reinforce the endogenous Prdx6, we chose SFN because of its effectiveness in cytoprotection and in treating/postponing oxidative/aging disorders^25,48,50,51^. Eyes are maximally exposed to UVB radiation. Therefore, we examined whether treatment with SFN would abate the cellular injuries evoked by UVB stress. We used antisense of Prdx6 (As-Prdx6) to knock down Prdx6 in SRA-hLECs as reported previously^7^. These transfectants were treated with SFN and then exposed to UVB and measured for viability and ROS production (Fig. 8A,B; lined bars). Cell viability assay revealed that SFN was significantly less effective in protecting SRA-hLECs having As-Prdx6. Also quantification of ROS levels in these SRA-hLECs showed that SFN did not lower ROS expression significantly (Fig. 8B, lined bars), suggesting that SFN acted mainly through Prdx6. In experiments to examine the cytoprotective ability of SFN against UVB-induced LECs injuries, we used SRA-hLECs, hLECs of different age groups and rLECs and pretreated them with SFN as indicated. Figure 8A,C and E show enhanced viability of SRA-hLECs, hLECs and rLECs (open vs gray and black bars; gray vs black bar) and reduced expression of ROS (Fig. 8B,D,F, open vs gray and black bars; gray vs black bar) with variable levels of UVB exposure (400 J/m^2^ or 800 J/m^2^) after SFN treatment. Data were normalized with absorbance of untreated controls. At concentration of 6 μM for hLECs and 2.4 μM and 4.8 μM for rLECs, SFN was effective in protecting LECs (Fig. 8). Moreover, none of the concentrations provided absolute protection, suggesting the involvement of other antioxidants augmented by SFN. Because other antioxidants did not protect hLECs against UVB stress significantly, we think that their protective role in lens/LECs may be minor compared to that of Prdx6.

## Discussion

In this study, we showed for the first time that, in aging, increased oxidative stress in lenses and lens cells is associated with failure of protective response due to dysregulation of Nrf2 and its target antioxidant gene Prdx6, and that this process was attenuated by application of SFN. Our work also revealed that ROS increase progressively during aging, and in aged cells become even more substantially increased (Fig. 1A), in a process directly related to progressive reduction in Nrf2,Prdx6 and Cat expression (Fig. 1B–D). Our data are consistent with results reported in other model systems^25,52,53^, showing the negative effects of aging on DNA binding activity of Nrf2^54^. This is thought to be caused by impaired cytoplasmic–nuclear shuttling of Nrf2 and age-related reduction in cellular abundance and activity of Nrf2^13,55–58^. Moreover, recently it was reported that Nrf2 levels are regulated by glycogen synthase kinase 3 (GSK-3) in Keap-independent pathways. Under strong oxidative stress, GSK-3 targets Nrf2 for β-TrCP-mediated proteasomal degradation, and thus the stability of Nrf2 is controlled via GSK-3/β-TrCPd^46,59^. We believe that strong activation of GSK-3 during normal aging or increased oxidative stress can lead to β-TrCP-mediated degradation of Nrf2, followed by repression of its target cytoprotective genes. Our DNA binding experiment with ARE probe derived from hPrdx6 promoter showed a significant reduction in Nrf2/ARE interaction in aging, and we noticed a dramatic reduction in Nrf2/ARE binding with nuclear fraction of elderly hLECs (Fig. 2A,B). Promoter assay also demonstrated that reduced binding of Nrf2 negatively affected Prdx6 transcription (Fig. 2C). Unfortunately, due to a scarcity of human samples (lenses) and limited proliferation of LECs in culture, we were not able to use the same hLECs for all the experiments, nor could we obtain samples with matched ages. Nonetheless, our study demonstrated an underlying molecular mechanism of Prdx6 repression that can be associated with a decline in Nrf2 expression/activity in aging lenses.

In previous studies, we found that Prdx6 depletion causes increased susceptibility to UVB- or H_2_O_2_-induced cell death. We also observed that other antioxidants were not effective at protecting LECs/lenses^7–9^, suggesting that Prdx6 expression is critical for protection of eye lenses. Prdx6 provides cytoprotection by removing ROS through its GSH peroxidase activity. ROS are produced in cells continuously through nonenzymatic and enzymatic reactions such as superoxide-dismutase (SOD)-catalyzed disproportionation of the superoxide radicals (O_2_^**•−**^) to H_2_O_2_ as well as by redox cycling. Continuous exposure to oxidants can also contribute substantially to the cellular steady state levels of H_2_O_2_ and O_2_^•**−**^. However, constitutive generation of H_2_O_2_ is derived mostly from mitochondria dependent upon NADH^60,61^ and activity of nicotinamide adenine dinucleotide phosphate (NADPH) oxidase (Nox) enzymes dependent on NADPH^62,63^. The primary function of NADPH oxidases is to produce ROS. These enzymes have been suggested to contribute to initiation of many diseases linked to oxidative stress^64^. Levels of ROS can also be raised during increased activity of cells and oxidative stress. Nonetheless, H_2_O_2_ is produced not only by mitochondria, but also by endoplasmic reticulum, peroxisomes and plasma membrane. Prdx6 is localized in these organelles, indicating the importance of this molecule in controlling the redox active state of cells.

Previously we found that Prdx6 expression declines with aging^1^. We surmised that the best strategy in this situation might be to use a natural activator such as SFN to restore the activity of Nrf2 and its target gene Prdx6. SFN preparations have been approved as clinically safe for use in healthy volunteers^65^. To ascertain whether SFN would induce the Nrf2/Prdx6 pathway in LECs, we examined levels of three antioxidants, Prdx6, Cat and GST*π*, in SRA-hLECs and rLECs. We specifically selected GST*π* as it is an electron donor to Prdx6, which controls the redox active state of Prdx6^66^. Figures 3 and 4 show that SFN induced expression of all three genes, Prdx6, Cat and GST*π* in dose-dependent fashion in both SRA-hLECs and rLECs. Closer *in silico* analysis of rat Prdx6 promoter (−10K from ATG sequences) revealed that it has several ARE-like sequences (data not shown), demonstrating that upregulation in antioxidant genes in rLECs occurred through the Nrf2 pathway. Surprisingly, our study revealed that SFN-treated SRA-hLECs displayed increased expression of GST*π*. This result, however, was not in agreement with a previously published report^67,68^. Our knockdown experiment with Sh-Nrf2 showed that SFN does activate GST*π* expression as shown, but not through Nrf2 (Supplementary Fig. 1). The different outcomes may be related to different cell types, or possibly SFN regulated GST*π* through pathways other than Nrf2/Keap1. The latter situation might be anticipated, since SFN has been shown to affect a number of pathways aside from Nrf2/Keap1 signaling^69^. There have been reports that GST*π* promoter bears ARE, Sp1 and ARE/TRE sites, which are required for its transcription^70,71^. Possibly, failure of Nrf2 to activate GST*π* in hLECs is due to the existence of Bach1 repressive signaling; Bach1 inactivation is required for GST*π* expression^72^. Furthermore, other transcription factors may be involved, like Sp1^72,73^, which may be activated through SFN^74^ and modulate GST*π* in hLECs. Nevertheless, further study is warranted to examine these possibilities.

Intriguingly, SFN-treated LECs displayed increased expression of Nrf2 mRNA and increased abundance of nuclear Nrf2 (Fig. 5), and this cellular response was time- and concentration-dependent. This observation is in agreement with a previous study showing that Nrf2 gene expression itself is regulated via ARE/Nrf2 mechanism^45^. Furthermore, our experiment demonstrated that SFN reinforced the nuclear accumulation of Nrf2 in LECs (Fig. 5B,C), and thereby enhanced Nrf2/ARE binding (Figs 6 and 7). We found that increased Nrf2/ARE activity was associated with increased promoter activity of Prdx6. However, mutant Prdx6 promoter retained some activity. The modulation in ARE-dependent gene transcription may be affected by Nrf2 interacting with proteins as it interacts with Jun/Fos family, Fra, small Maf, and ATF4^13,47^. These factors may modify the transcription potential of Nrf2 in activating ARE-mediated transcription.

UVB is a major culprit for inducing oxidative damage of eye lens/LECs. We found that rLECs and SRA-hLECs pretreated with SFN showed resistance against UVB injuries (Fig. 8A–D). In addition, SFN protected aging hLECs against UVB stress. We think that SFN does so by activation of the Nrf2/ARE pathway as evidenced by Figs 6 and 7. Examining the contribution of Prdx6 in rescuing SFN-treated LECs, our Prdx6-knockdown experiment revealed that SFN without Prdx6 became significantly less effective in protecting LECs facing UVB (Fig. 8A,B). Other antioxidants that were reinforced by SFN failed to protect LECs, indicating that Prdx6 is essential to protect LECs against UVB. Moreover, we also recognize that oxygen levels in the eye are generally low, as maintaining lens clarity over a prolonged time is essential^75,76^. Nevertheless, hLECs are metabolically highly active, with high concentrations of mitochondria. ROS generation due to oxygen or reductive stress-induced ROS (in the hypoxic range) is slowed through an enriched antioxidant defense system of LECs that can normalize functioning and thereby maintains lens homeostasis (adaptive response). Thus we think that LECs cultured *in vitro* behave similarly to other cells due to adaptive responses. Several published studies as well as our own study have tested antioxidant activity of biochemical reagents with *in vitro* model systems (20% O_2_) by applying exogenous stresses, and these activities have been reproduced *in vivo* ^8,77–81^. However, the *in vitro* study conducted in the current research should clarify Prdx6′s ability to protect LECs and its regulation by SFN.

In summary, we have shown that Nrf2 and its mediated genes are dysregulated in aging LECs and lenses. Importantly, activation of Nrf2 can be reinforced by treating aged lens cells with SFN. The study also detailed the molecular mechanism that occurs during aging, at least in lens/LECs, i.e., the increased accumulation of oxidative load due to failure of antioxidant response, and found that Prdx6 expression is required to reverse the pathogenic process in LECs. Based upon this work, we propose a chemopreventive strategy of using small molecules like SFN to block/delay cataractogenesis or etiopathogenesis in eye lens.

## Methods

### Cell culture and treatments

Primary rat LECs (rLECs) were isolated from 6-week-old Sprague-Dawley albino rats (n = 8) as described previously^8^. The rLECs were maintained in Dulbecco’s Modified Eagle’s Media (DMEM; Life Technologies, Carlsbad, CA, USA) with 10% fetal bovine serum (FBS; Sigma, St. Louis, MO, USA). rLECs reaching 80 to 90 percent confluence were harvested and used for assays. All the experiments on rLECs were conducted at passages (P) 3 to 5. Isolation of rLECs from animals was approved by the Kanazawa Medical University, and procedures were conducted in accordance with the National Institutes of Health Guidelines for Laboratory Animals at the Kanazawa Medical University, Japan.

Human LECs used were of two types: (1) a cell line (SRA01/04) immortalized with SV40, and (2) primary human LECs isolated from deceased persons of different ages. To avoid confusion, the remaining text will designate the immortalized LECs as SRA-hLECs, and the primary human (h) LECs as primary hLECs or hLECs

The SRA-hLECs were derived from 12 infants who underwent surgery for retinopathy of prematurity^82^ (a kind gift of Dr. Venkat N. Reddy, Eye Research Institute, Oakland University, Rochester, MI, USA). These cells were maintained in DMEM with 15% FBS, 100 µg/ml streptomycin, and 100 µg/ml penicillin in 5% CO_2_ environment at 37 °C as described previously^83,84^.

### Isolation and generation of hLECs

Primary hLECs were isolated from normal eye lenses of deceased persons or healthy donors of different ages (16, 18, 21, 24, 26, 34, 36, 52, 56, 58, 62, 64, 65, 66, 68 and 75 y) obtained from the Lions Eye Bank, Nebraska Medical Center, Omaha, NE, USA and National Development & Research Institute (NDRI), Inc., PA, USA. According to regulation HHS45CFR 46.102(f), studies involving material from deceased individuals are not considered human subject research as defined at 45CFR46.102(f) 10(2) and do not require IRB oversight. To conduct our work successfully due to limited sample size, eye lenses were divided into groups by age: lenses age and number 16, 18 and 21 y, n = 6; ages 24, 26 and 26 y, n = 6; ages 34 and 36 y, n = 4; ages 52, 56 and 58 y, n = 6; ages 62, 62, 64, 65, 66 and 68 y, n = 12; and ages 75 and 75 years, n = 4. For RNA expression and DNA interaction studies, lenses used from each group for this purpose were those aged 16, 26 34, 36, 52, 62, 65, 66 and 75 y. The remaining lenses were used for generation of LECs for other experiments mentioned in this study. Briefly, the capsule was trimmed before explanting in 35mm culture dishes precoated with collagen IV containing a minimum amount of DMEM containing 15–20% fetal bovine serum (FBS), with a brief modification^8,9,85,86^. Capsules were spread by forceps with cell layers upward on the surface of plastic petri dishes. Culture explants were trypsinized and re-cultured. Cell cultures attaining 90 to 100 percent confluence were trypsinized and used for experiments^49,84,87^. Western analysis was used to validate the presence of αA-crystallin, a specific marker for LEC identity (data not shown). For the experiments, SRA-hLECs and/or hLECs were cultured in 96, 24, 48 or 6 well plates or 60 and 100 mm petri dishes according to the specific requirements of each experiment. To examine the effect of SFN (1-isothiocyanato-4(methylsulfinyl)-butane, Cat. No. S4441, Sigma-Aldrich, St Louis, MO, USA), cells were treated with different concentrations of SFN (rLECs: 2.4, 4.8, 9.6 and 19.2 µM and SRA-hLECs: 1, 3, 6, 8 or 12 μM) for variable time intervals.

### Cell survival assay (MTS assay)

A colorimetric MTS assay (Promega, Madison, WI, USA) was performed as described earlier^8,9,88^. This assay of cellular viability uses 3-(4,5-dimethylthiazol-2-yl)-5-(3-carboxymethoxyphenyl)-2 to 4-(sulphophenyl) 2H-tetrazolium salt. The A_490_ nm (O.D.) value was measured after 2 h with a plate reader, Spectra Max Gemini EM (Mol. Devices, Sunnyvale, CA). Results were normalized with absorbance of the untreated control(s).

### Quantitation of ROS levels by H2-DCF-DA assay

SRA-hLECs or primary hLECs or rLECs were cultured in 96 well plates (5 × 10^3^/well) in the presence or absence of SFN. At predefined times these cells were subjected to UVB stress. After eight hours levels of ROS were measured by using fluorescent dye dichlorofluorescin diacetate (H2-DCF-DA), a nonpolar compound that is converted into a polar derivative (dichlorofluorescein) by cellular esterase after incorporation into cells^7^. Levels of ROS (intracellular fluorescence) were detected at excitation (Ex) 485 nm/emission (Em) 530 nm by Spectra Max Gemini EM (Mol. Devices, Sunnyvale, CA).

### Real-Time Reverse Transcriptase-Polymerase Chain Reaction (RT-PCR)

The total RNA from the SRA-hLECs, rLECs and primary hLECs directly detached from lenses (to avoid cell culture effect) was extracted using RNeasy Mini Kit (Qiagen, Valencia, CA) following the manufacturer’s protocol. Total RNA from lenses of variable ages was extracted to examine the levels of Nrf2 and Prdx6. From 0.5 to 2 micrograms of total RNA was reverse-transcribed with High Capacity cDNA Reverse Transcription Kit following the manufacturer’s instructions. For rLECs, a Gene Amp PCR System 9700 (Applied Bio Systems, Foster City, CA, USA) was used; for hLECs and SRA-hLECs the SYBR Green Master Mix (Roche Diagnostic Corporation, Indianapolis, IN) in a Roche® LC480 Sequence detector system (Roche Diagnostic Corporation) was employed. GST*π*, Catalase, and Prdx6 as well as Nrf2 gene expressions were analyzed with RT-PCR on 7300 Real Time PCR System (Applied Biosystems) using the primers designed for each molecule of rat genes (TaqMan; ratPrdx6 probe ID: Rn01759191_g1; rat catalase probe ID: Rn00560930_m1; rat GST*π* probe ID: Rn00561378_gH) or human genes (Universal probe library for human; Prdx6 probe ID: NM_004905.2; catalase probe ID:NM_001752.3; GST*π* probe ID:NM_000852.3; Nrf2 probe ID: NM_001145413 and β-actin probe ID: NM_001101.3). 18 S ribosomal RNA (Applied Biosystems), β-actin as an endogenous control, and/or both were used to normalize the expression of GST*π*, Catalase and Prdx6 in each group. The relative quantity of mRNA was obtained using the comparative CT method.

### Protein expression analysis

Cell extract of LECs were prepared in ice-cold radioimmune precipitation buffer and protein blot analysis was performed as described previously^1,89^. The membranes were probed with Anti-Prdx6 antibody (Ab) (Abcam^®^, Cambridge, MA, USA and Lab Frontier, Seoul, Korea), anti-catalase antibody (Sigma-Aldrich and Santa Cruz Biotechnology), anti-GST*π* Ab (Abcam^®^) or anti-Nrf2 antibody (Stressgen Bioreagents Corp, Victoria, BS, CA, USA and Abcam and Santa cruz, USA) or β-actin (internal control, Sigma-Aldrich) and tubulin (Abcam) to monitor those protein expressions. After secondary antibody treatment, protein bands were visualized by incubating the membrane with luminol reagent (sc-2048; Santa Cruz Biotechnology). Images were recorded with a FUJIFILM-LAS-4000 luminescent image analyzer (FUJIFILM Medical Systems Inc., Hanover Park, IL, USA).

### Extraction of nuclear and cytosolic fraction

Nuclear extracts from LECs were prepared as described earlier^9,90^. Briefly, cells were cultured in 35 or 60 or 100mm plates. Cells were suspended in cytoplasmic extraction buffer (10 mM Hepes, 60 mM KCL, 1 mM EDTA, 0.075%[v/v] NonidetP-40,1 mM phenylmethylsulfonyl fluoride, adjusted to pH 7.6). After cells were washed with cytoplasmic extract buffer without detergent (Nonidet P-40), fragile nuclei were re-suspended in nuclear extract (NE) buffer (20 mM Tris-HCl, 420 mM NaCl, 1.5 mM MgCl_2_, 0.2% EDTA, 1 mM phenylmethylsulfonyl fluoride, and 25% [v/v] glycerol, adjusted to pH 8.0). The salt concentration was adjusted to 400 mM, and the nuclear faction was incubated on ice for 2 h with vortexing. After dialysis, protein concentration was estimated according to the Bradford method and used for assays.

### Gel-shift and depletion assays

Oligonucleotides containing Nrf2 binding site derived from Prdx6 gene promoter or its mutant at ARE were synthesized (Invitrogen). Sequences were annealed and labeled with [ϒ-^32^P] ATP using T4 polynucleotide kinase (New England Biolabs, Inc.). The binding reaction was performed in 20 µl buffer containing 20 mM Tris-HCl (pH 8.0), 75 mM KCL, 5% glycerol, 50 µg/ml bovine serum albumin (BSA), 0.025% nonidet NP-40, 1 mM EDTA, 5 mM DTT, and 1 µg of poly (dI/dC). The labeled probe (5fmol [1000 cpm]) was incubated on ice for 30 min with 5 µg or 10 µg of nuclear extract was isolated from SRA-hLECs as well as from hLECs directly detached from the lenses to avoid cell culture effects (Fig. 2). Samples were loaded on a 5% polyacrylamide gel in 0.5XTBE buffer and autoradiographed. In competition assays, a 1000-fold molar excess of cold probe was added. For depletion assay, nuclear extracts were incubated with either anti-Nrf2 antibody (SantaCruz Biotech, Santa Cruz, CA) or normal rabbit IgG, at 4 °C O/N and extract was used in gel-shift assay.

### Construction of human Prdx6 promoter-chloramphenicol acetyltransferase (CAT) reporter vector

The 5′-flanking region (−918 to +30 bp) was isolated from human genomic DNA by using an Advantage® Genomic PCR Kit (Cat. No. 639103 &639104, Clontech Laboratories, Inc, Mountain View, CA 94043). The product obtained was cleaned and sequenced as described previously^84,91^. A construct of −918 bp was prepared by ligating it to basic pCAT vector (Promega) using the *SacI* and *XhoI* sites. The plasmid was amplified and sequenced. Primers were as follows: Sense; 5′-GACAGAGTT*GAGCTC*CACACAG-3′; and antisense; 5′-CACGTC*CTCGAG*AAGCAGAC-3′.

### Site-directed mutagenesis (SDM)

PCR-based site-directed mutagenesis was carried out using the QuikChange^TM^ lightning site-directed mutagenesis kit (Agilent Technologies; Catalog No. 210518), following the company’s protocol. Briefly, amino acid exchanges at the Nrf2 site (ARE; −357/−349) mutant (TG to GT) were generated by point mutations in the human promoter of Prdx6-CAT construct. The following complementary primers were used (changed nucleotides are in boldface type and underlined):

Nrf2-Mut_for_ 5′-CCAGGGGGCAACG**GT**ACCGA*GC*CCCGCATCACGTGTGC-3′;

Nrf2-Mut_rev_ 5′-GCACACGTGATGCGGG*GC*TCGGT**AC**CGTTGCCCCCTGG-3′.

Epicurean Coli XL1-Blue super-competent cells (Stratagene) were transformed with resultant plasmid. The plasmid was amplified, and the mutation was confirmed by sequencing as described previously^91^.

### Transactivation assay

A chloramphenicol acetyltransferase (CAT)-enzyme-linked immunosorbent assay (ELISA) kit (Roche Applied Science) was used as described in our previously published protocol^49,91^. SRA-hLECs or primary hLECs of variable ages were transfected/co-transfected with Prdx6 (−918/+30 bp)-CAT reporter plasmid or CAT empty vector (1, 2 or 4 µg) along with pGFP (0.25 or 0.5 or 1 µg) depending upon cell culture model. CAT-ELISA was performed and absorbance was measured at 405 nm. Transactivation activities were adjusted for transfection efficiencies using GFP Optical Density (O.D.) values recorded at EX485/Em530nm.

### Chromatin Immunoprecipitation (ChIP) Assay

ChIP was performed using the ChIP-IT® Express (Cat. No. 53008; Active Motif, Carlsbad, CA, USA) and ChIP-IT® qPCR analysis kit (Cat. No. 53029; Active Motif, Carlsbad, CA, USA) following the manufacturer’s protocol^49^. Antibodies used were control IgG and antibody specific to Nrf2 (Abcam, Cat. No. Ab62352). Real-time PCR amplification was carried out using 5 μl of DNA sample with primers (human Prdx6 promoter bearing Nrf2 site [ARE; −357/−349]), forward primer: 5′-CAGAGTCAAACCTGGCGCATC-3′ and reverse primer: 5′-CATCCTTCAGACACTATAGGCC-3′ specific to the Prdx6 promoter. The program for quantification amplification was 2 min at 95 °C, 15 s at 95 °C, 20 s at 58 °C and 20 s at 72 °C for 40 cycles in 20 μl reaction volume. Data wer plotted and presented in the form of a histogram.

### Induction of ultraviolet (UV) B induced stress

For UVB treatment, rLECs, SRA-hLECs or primary hLECs were pre-cultured for 24 h in a 96 well-plate with DMEM-10% or 15% FBS with predefined concentrations of SFN. The medium was replaced with phosphate buffered saline (PBS, pH 7.2) and the plates containing the monolayers were exposed to 0, 400 J/m² or 800 J/m² UVB using UV-lamp emitting 270–320 nm peaking at 302 nm wavelength (UVP, Upland, CA, USA). The energy actually incident onto the working area was measured by a UVX Radiometer (UVP Inc., Upland, CA) and expressed in J/m^2^. The UV dosage of J/m^2^ (0, 100 or 200 sec exposure time) was selected on the basis of results from our previous work^92^. After irradiation, PBS was withdrawn and fresh medium was added. Eight and twenty-four hours later ROS and MTS assays were performed to monitor the levels of ROS and cell viability, and the percentage of ROS and cell survival levels was then calculated for each group, respectively.

### Construction of Prdx6 antisense

A human LEC cDNA library was used to isolate Prdx6 cDNA having a full-length open reading frame. A full-length Prdx6 antisense (Prdx6-As) construct was made by sub-cloning Prdx6 cDNA into a pcDNA3.1/NT-GFP-TOPO vector in reverse orientation. Plasmid was amplified following TOP 10 bacterial cells transformation as described earlier^9^.

### Statistical methods

For all quantitative data collected, statistical analysis was conducted by Student’s *t test* and/or one-way ANOVA when appropriate, and was presented as mean ± S.D. of the indicated number of experiments. A significant difference between control and treatment group was defined as *P* value of < 0.05 and 0.001 for two or more independent experiments.

## Electronic supplementary material

Supplementary Information
