# Supplementary material for: Sulforaphane reactivates cellular antioxidant defense by inducing Nrf2/ARE/Prdx6 activity during aging and oxidative stress
Source: Sci Rep. 2017 Oct 26;7:14130. doi: 10.1038/s41598-017-14520-8 (PMC5658327; doi:10.1038/s41598-017-14520-8)
Supplement: Supplementary file 1 — Supplementary Information [file 41598_2017_14520_MOESM1_ESM.pdf]

Sulforaphane reactivates cellular antioxidant defense by inducing Nrf2/ARE/Prdx6 activity during aging and oxidative stress

Eri Kubo[1]*, Bhavana Chhunchha[2], Prerna Singh[2], Hiroshi Sasaki[1], Dhirendra P Singh[2#]

[1]Department of Ophthalmology, Kanazawa Medical University, Japan

[2]Department of Ophthalmology and Visual Science, University of Nebraska Medical Center, Omaha NE, USA

Short title: Sulforaphane repairs Nrf2/Prdx6 dysregulation

Correspondence:

Japan: *Eri Kubo, MD, PhD, Professor, Department of Ophthalmology, Kanazawa Medical University, 1-1 Daigaku, Uchinada, Kahoku, Ishikawa 920-0293, Japan

Tele: +81-76-286-2211; Fax: +81-76-286-1010; Email: kuboe@kanazawa-med.ac.jp

USA: #Dhirendra P. Singh, PhD, Professor, Department of Ophthalmology and Visual Sciences, University of Nebraska Medical Center, Omaha NE 68198-5840, USA

Tele: 1-402-559-8805; Fax: 402-559-8808; Email: dpsingh@unmc.edu

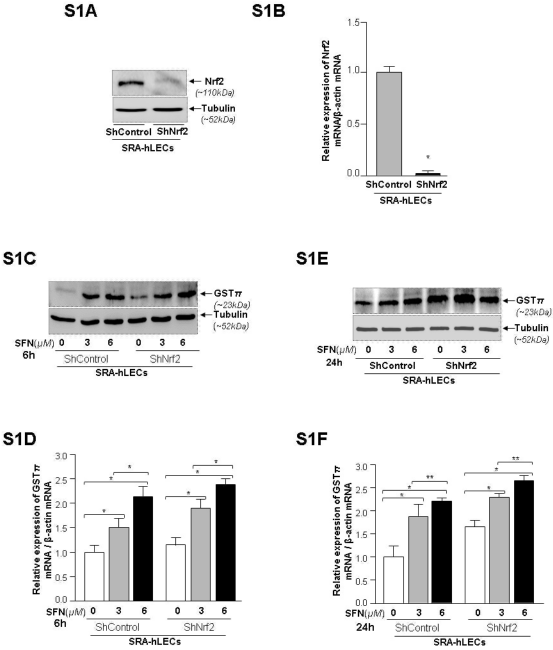
S1A
Nrf2
(~110kDa)
Tubulin
(~52kDa)
ShControl ShNrf2
SRA-hLECs
S1B
Relative expression of Nrf2 mRNA/β-actin mRNA
1.5
1.0
0.5
0.0
*
ShControl ShNrf2
SRA-hLECs
S1C
GSTπ
(~23kDa)
Tubulin
(~52kDa)
SFN(μM) 0 3 6 0 3 6
6h
ShControl ShNrf2
SRA-hLECs
S1E
GSTπ
(~23kDa)
Tubulin
(~52kDa)
SFN(μM) 0 3 6 0 3 6
24h
ShControl ShNrf2
SRA-hLECs
S1D
Relative expression of GSTπ mRNA / β-actin mRNA
3.0
2.5
2.0
1.5
1.0
0.5
0.0
SFN(μM) 0 3 6 0 3 6
6h
ShControl ShNrf2
SRA-hLECs
S1F
Relative expression of GSTπ mRNA / β-actin mRNA
3.0
2.5
2.0
1.5
1.0
0.5
0.0
SFN(μM) 0 3 6 0 3 6
24h
ShControl ShNrf2
SRA-hLECs

S1A

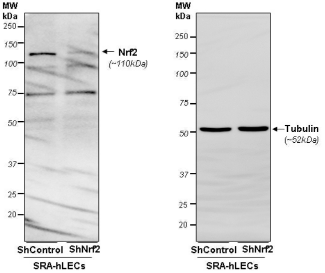
MW
kDa
250
150
100
75
50
37
25
20
Nrf2
(~110kDa)
ShControl
ShNrf2
SRA-hLECs
Tubulin
(~52kDa)

S1C

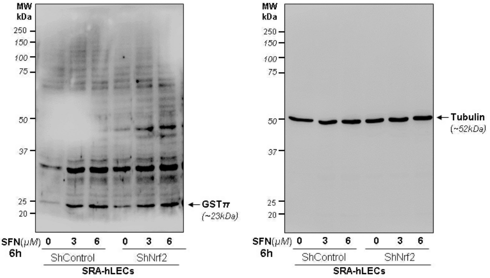
MW
kDa
250
150
100
75
50
37
25
20
GSTπ
(~23kDa)
SFN(μM) 0 3 6 0 3 6
6h
ShControl
ShNrf2
SRA-hLECs
Tubulin
(~52kDa)

S1D

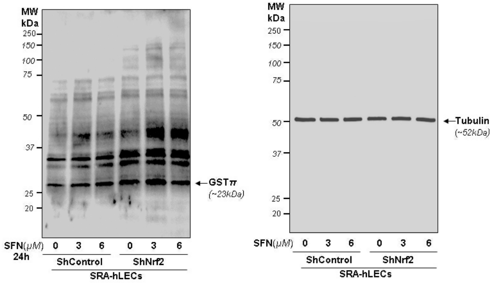
MW
kDa
250
150
100
75
50
37
25
20
GSTπ
(~23kDa)
SFN(μM) 0 3 6 0 3 6
24h
ShControl
ShNrf2
SRA-hLECs
Tubulin
(~52kDa)

Fig. 3C

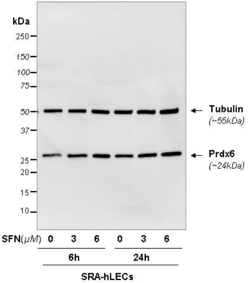

Fig. 3E

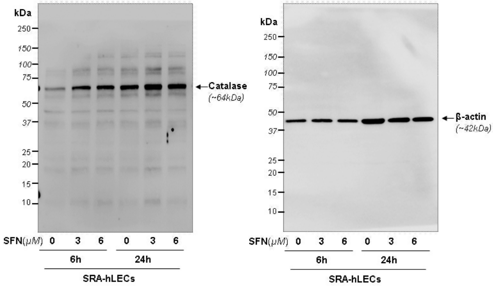

Fig. 3G

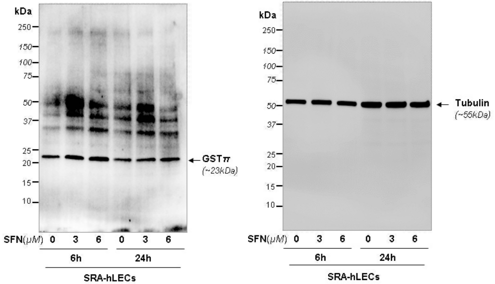

Fig. 4C

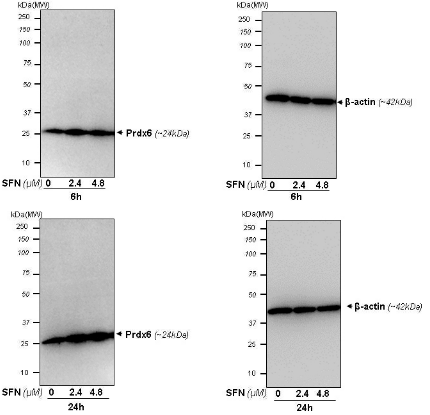
kDa(MW)
250
150
100
75
50
37
25
10
Prdx6 (~24kDa)
SFN (μM) 0 2.4 4.8
6h
β-actin (~42kDa)
24h

Fig. 5B

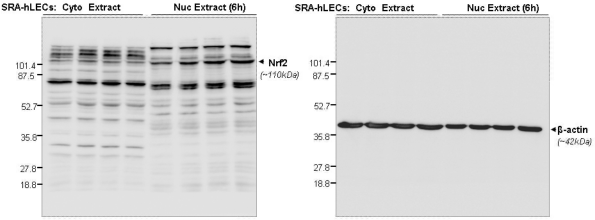

SRA-hLECs: Cyto Extract
Nuc Extract (6h)
101.4
87.5
52.7
35.8
27.8
18.8
Nrf2
(~110kDa)
SRA-hLECs: Cyto Extract
Nuc Extract (6h)
101.4
87.5
52.7
35.8
27.8
18.8
β-actin
(~42kDa)

Supplementary Methods

Nrf2 knockdown with Nrf2-shRNA

Nrf2 expression in SRA-hLECs was silenced by using Nrf2-shRNA (ShNrf2) plasmid (h) (sc-37030-SH, Santa Cruz Biotechnology). Transfections were carried out with the Neon transfection system (Invitrogen). $2X10^6$ cells were transfected with 2µg control shRNA (ShControl) and shNrf2 for 48h, and transfectants expressing corresponding shRNA were selected by puromycin (5µg/ml). Puromycin containing DMEM culture media was replaced every 48h, until resistant colonies could be identified (8 to 10 days). ShControl plasmid (sc-108060) was used as control. The expressions of targeted mRNA, protein and loading control in stable cells were verified by real-time PCR and Western analysis using probe specific to Nrf2. Selected transfectants ($4X10^5$) were cultured in 60mm culture dishes overnight. The next day, they were treated with DMSO or 3µM or 6µM of SFN for 6h and 24h. Total RNA and protein were isolated and processed for mRNA and Western analysis using probe specific to Nrf2.

Legends

Supplementary Figure 1

Figures. S1A and S1B, Representative Immunoblots and mRNA analysis showing depletion of Nrf2 using Nrf2 Knockdown assay. Nrf2-specific shRNA plasmid was transfected and selected as described in the supplementary Methods section. Cellular lysate and total RNA were isolated, followed by Western analysis and real-time PCR. The same membrane was probed and reprobed with antibodies following stripping and restripping to obtain relative expression of Nrf2 and tubulin expression. **S1C to S1F**, Increased GSTπ expression levels in SFN treated shControl and Nrf2 knockdown in SRA-hLECs. $4X10^5$ shControl and Nrf2 knockdown SRA-hLECs were cultured in 60mm culture dishes overnight. Cellular lysate and RNA were isolated and processed for Western analysis and real-time PCR using probes specific to GSTπ to measure the expression levels of GSTπ as indicated. S1D and S1F, *p<0.001 and **p<0.05.

Supplementary Figure 1A, 1C and 1E

Figures. S1A, **Representative full Immunoblots analysis showing depletion of Nrf2 using Nrf2 Knockdown assay.** Nrf2-specific shRNA plasmid was transfected and selected as described in the supplementary Methods section. Cellular lysate were isolated, followed by Western analysis. The same membrane was probed and reprobed with antibodies following stripping and restripping to obtain relative expression of Nrf2 and tubulin expression. **S1C and S1E**, Increased GST*π* expression levels in SFN treated shControl and Nrf2 knockdown in SRA-hLECs. $4X10^5$ shControl and Nrf2 knockdown SRA-hLECs were cultured in 60mm culture dishes overnight. Cellular lysate were isolated and processed for Western analysis using probes specific to GST*π* to measure the expression levels of GST*π* as indicated. MW; Molecular weight marker in kDa.

Figure 3C, 3E and 3G. SFN enhanced expression of antioxidants Prdx6, Cat and Phase II protein GST*π* protein in dose-dependent manner in SRA-hLECs. (**C, E and G**) SFN significantly enhanced Prdx6, Catalase and GSTπ protein expression. Cells were treated with DMSO vehicle or different concentrations of SFN for 6h and 24h. Total protein were extracted, and subjected to immunoblotting using specific probes as indicated. SFN produced a concentration-dependent increased pattern of Prdx6 (C), Catalase (E) and GST*π* (G) protein expression. β-actin and tubulin used as internal control. MW; Molecular weight marker in kDa.

Figure 4C. Rat LECs treated with SFN displayed increased levels of antioxidant gene/protein Prdx6. rLECs treated with SFN showed enhanced expression of protein. Cells were treated with 2.4μM or 4.8μM of SFN or DMSO as indicated. Total protein were isolated and process for Western analysis with Prdx6 specific probes revealed a concentration-dependent increased expression of Prdx6 protein (C). β-actin were used as loading control. MW; Molecular weight marker in kDa.

Figure 5B. SFN induced Nrf2 expression and enhanced nuclear accumulation in both SRA-hLECs. (**B**) SFN-mediated induction of Nrf2 expression and nuclear localization. Cultured SRA-hLECs were treated with different concentrations of SFN for 6h. Cytosol and nuclear extract were immunoblotted with anti-Nrf2 antibody. β-actin was used as loading control. MW; Molecular weight marker in kDa.
